# Early Skin Biopsy in Conradi‐Hünermann‐Happle Syndrome (X‐Linked Dominant Chondrodysplasia Punctata)

**DOI:** 10.1111/cup.14837

**Published:** 2025-07-09

**Authors:** Cathal O'Connor, Neidín Bussmann, Sarah Ni Mhaolcatha, Cynthia Heffron, Sally O'Shea

**Affiliations:** ^1^ Dermatology South Infirmary Victoria University Hospital Cork Ireland; ^2^ Neonatology Cork University Maternity Hospital Cork Ireland; ^3^ Medicine University College Cork Cork Ireland; ^4^ Pathology Cork University Hospital Cork Ireland

**Keywords:** blaschkolinear, erythroderma, genodermatosis, ichthyosis, neonates

Conradi‐Hünermann‐Happle syndrome (CHHS) or X‐linked dominant chondrodysplasia punctata (CDPX2, OMIM 302960) is a rare type of chondrodysplasia punctata associated with X‐linked dominant variants in the emopamil binding protein (*EBP*) gene, which impairs cholesterol biosynthesis [1]. The syndrome is characterized by the triad of ichthyosis (usually presenting as congenital ichthyosiform erythroderma) in 95%, skeletal dysplasia in 80%, and congenital cataracts in 60% [1]. Scarring alopecia can also be present [2]. Dystrophic calcifications in keratotic follicular plugs represent a unique histopathologic feature of CHHS in newborns that has not been noted in other forms of ichthyoses [3]. The characteristic congenital ichthyosiform eruption clears spontaneously within a few weeks, so early biopsy is essential to capture the corresponding diagnostic histopathologic features [3].

We report the case of a first‐born female infant who was delivered at term with widespread redness and scale. There was no family history of genodermatoses or inflammatory dermatoses. There was no parental consanguinity and no history of miscarriages. On examination, there was erythroderma, generalized thick adherent scale in a feathery pattern following lines of Blaschko, and shiny red whorls on the dorsolateral feet (Figure 1A–1F). There was no skin peeling or skin fragility. Several fingernails were hypoplastic. The nasal bridge was flat, and the neck was short. Red reflexes were present bilaterally, but both eyes were noted to be small. The head and trunk were large relative to the limbs, and the proximal limb segments were proportionately short.

**FIGURE 1:**
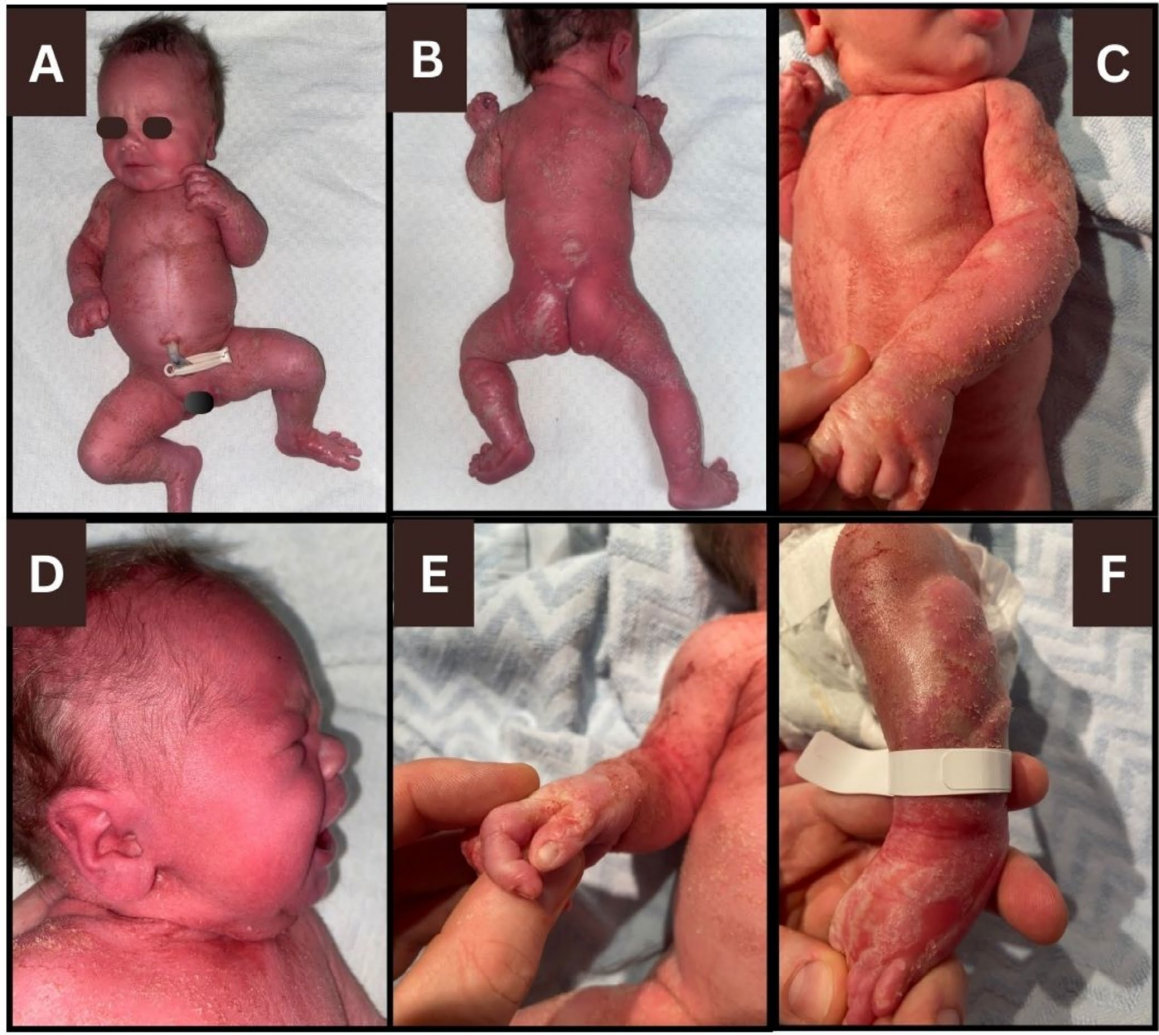
Clinical images taken in the first few hours of life showing erythroderma and generalized thick adherent scale in a feathery blaschkolinear pattern (Figure 1A–1C), flat nasal bridge (Figure 1D), nail dystrophy (Figure 1E), and shiny red whorl on the dorsolateral left foot (Figure 1F).

Given the constellation of signs, CHHS was suspected clinically. Skin biopsies in the first 12 h of life from linear scaly streaks showed orthohyperkeratosis and numerous dilated follicular ostia with keratin plugs (Figure 2A–2B). Foci of calcification were seen in the corneocytes of the stratum corneum and hair follicles, highlighted with a von Kossa stain (Figure 2C–2D). Skeletal survey showed symmetric punctate calcification/stippling of the proximal femoral epiphysis and ankle bilaterally and of the right humeral epiphysis and right carpus, in keeping with chondrodysplasia punctata, although no gross rhizomelia was appreciated radiologically. Ophthalmology review did not identify congenital cataracts. Renal and cranial ultrasounds were normal, and audiology was normal. Blood tests showed normal hematologic, renal, and bone parameters, and a hyperbilirubinemia meeting the phototherapy threshold. The erythema was noted to improve following 12 h of phototherapy. Urea 10% cream was helpful in reducing the scale. Single gene testing for a variant in the *EBP* gene was requested from blood, and a pathogenic variant was detected (c.184C>T; p.Arg62Trp), confirming the diagnosis of CHHS. Parental genetic testing for the variant was negative. On follow‐up at six weeks of age, her skin had completely normalized, with no evidence of erythema or ichthyosis. Orthopedic follow‐up is ongoing.

**FIGURE 2:**
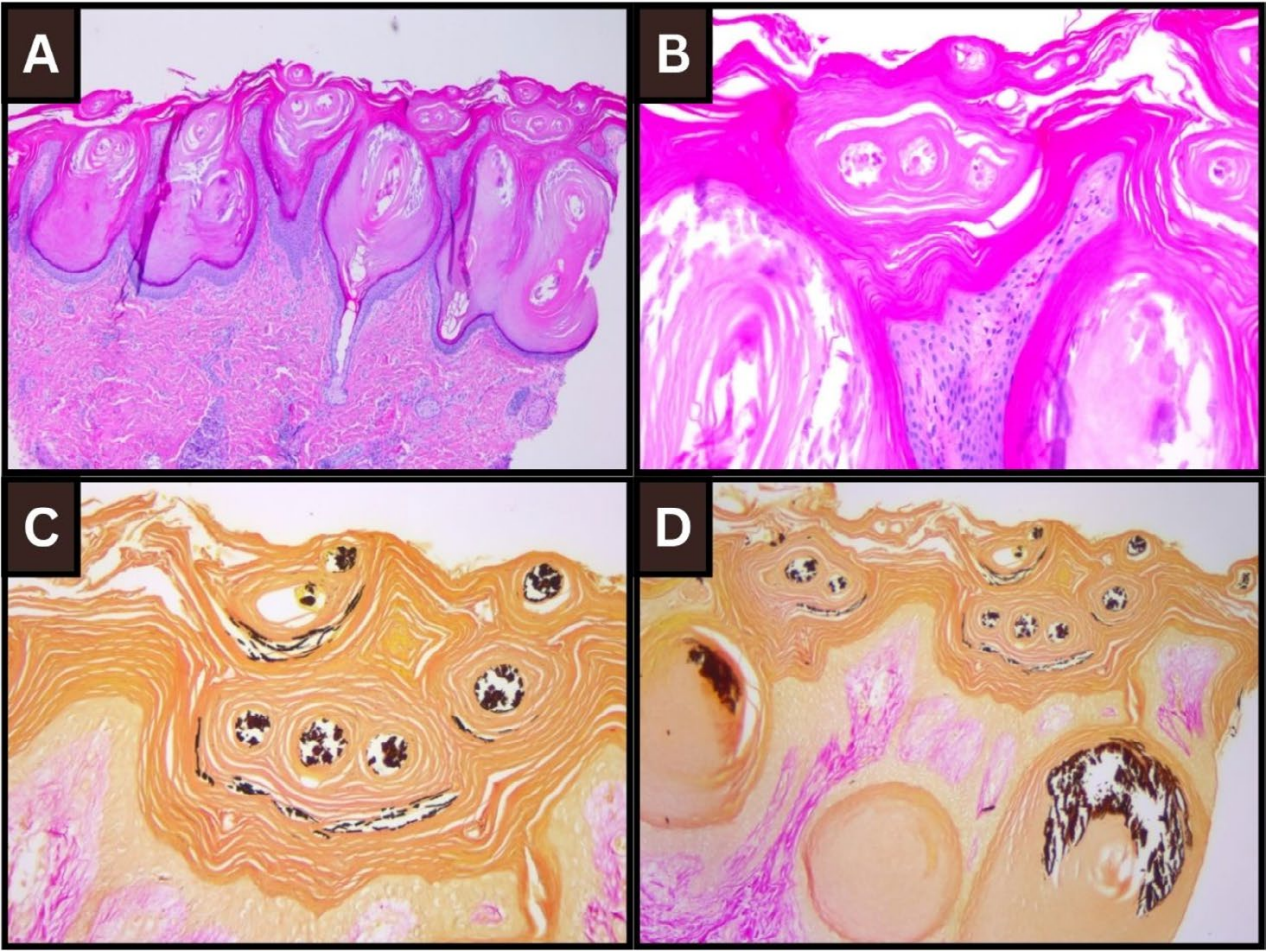
Histopathology of skin biopsies showing orthohyperkeratosis and numerous dilated follicular ostia with keratin plugs on low power (Figure 2A, 100× H&E) and high power (Figure 2B, 200× H&E); and foci of calcification in the corneocytes of the stratum corneum and hair follicles, highlighted with a von Kossa stain (Figure 2C, 200× von Kossa, and Figure 2D, 100× von Kossa).

The X‐linked dominant inheritance pattern of CHHS was first described by Happle in 1979 [4]. Affected unborn males are not expected to survive to birth, although there are reported cases in male patients with Klinefelter syndrome or mosaicism caused by an early postzygotic mutation in the *EBP* gene [5]. The cutaneous phenotype is variable and improves rapidly over the first few weeks of life, due to the effects of X‐inactivation in females [6]. In neonates, it is characterized by congenital ichthyosiform erythroderma, blaschkolinear “feathery” hyperkeratosis, rhizomelia, congenital cataract(s), midface hypoplasia, and a wide flat nose. Other dermatologic manifestations may include collodion membrane, cicatricial alopecia, persistent ichthyosis, follicular atrophoderma, pigmentary abnormalities, and nail dystrophy [5].

Although the pathology of CHHS has only been reported in a few cases, typical features include hyperkeratosis and dilated ostia of the pilosebaceous units [7]. Dystrophic calcifications within keratotic infundibular follicular plugs, especially in the stratum corneum, are a unique but under‐recognized histopathologic feature of newborns with CHHS [3, 7, 8, 9]. The mechanism of calcium deposition in the epidermis is unclear, although it may be related to the impact of the abnormal cholesterol on the Sonic, Desert, and Indian hedgehog pathways, which are known to be involved in both epidermal and bone homeostasis [8]. Calcium deposition on epidermal keratins is an extremely rare phenomenon and has only been reported in transepidermal elimination of calcium deposits in calcinosis cutis and in plantar corns [10].

It is important to perform the skin biopsy early, as the dystrophic calcifications typically resolve spontaneously after the first few weeks of life, in tandem with clinical resolution of the ichthyosiform erythroderma. After the ichthyosiform erythroderma has resolved, blaschkolinear hypopigmentation and follicular atrophoderma can develop. However, the distinctive dystrophic calcification will be no longer present, so later biopsy is less helpful diagnostically [1]. In this case, the clinical diagnosis was suspected immediately at birth, and the biopsy was performed in the first 12 h of life, which explains why all characteristic features were present so prototypically in the biopsy.

Other investigations to aid diagnosis in the neonatal period include sterol analysis [showing increased 8(9)‐cholestenol and 8‐dehydrocholesterol] in blood and genetic analysis (showing pathogenic variants in the *EBP* gene) [1]. The differential diagnosis for CHHS includes congenital hemidysplasia with ichthyosiform nevus and limb defects (CHILD) syndrome, another X‐linked dominant disorder caused by heterozygous loss of function mutations in *NSDHL*, which is associated with verruciform xanthoma and inflammatory and lipid‐laden infiltrates within the dermal papillae rather than intracorneal calcifications [11].

Although access to genetic testing has improved dramatically in recent years, and turnaround time has reduced significantly, the potential of early skin biopsy for histopathology to rapidly guide the diagnosis of rare genodermatoses such as CHHS in the neonatal period should not be forgotten.

## Ethics Statement

Ethical approval was not applicable.

## Conflicts of Interest

The authors declare no conflicts of interest.

## Data Availability

Data are available on request from the corresponding author.
